# Findings from a cluster randomised feasibility study of a school-based physical activity role model intervention (CHARMING) for 9–10-year-old girls

**DOI:** 10.1186/s40814-025-01615-7

**Published:** 2025-04-04

**Authors:** Kelly Morgan, Jordan Van Godwin, Rebecca Cannings‑John, Rhiannon Tudor Edwards, Rachel Granger, Britt Hallingberg, Graham Moore, Bethan Pell, Esther van Sluijs, Holly Whiteley, Jemma Hawkins

**Affiliations:** 1 Centre for Development, Evaluation, Complexity and Implementation in Public Health Improvement (Decipher), Cardiff University, Spark, Maindy Road, Cardiff, CF24 4HQ UK; 2 Centre for Trials Research, Cardiff University, Heath Park, Cardiff, CF14 4YS UK; 3 Centre for Health Economics and Medicines Evaluation, Bangor University, Normal Site, Holyhead Road, Bangor, Gwynedd LL57 2PZ UK; 4 Cardiff School of Sport and Health Sciences, Cardiff Metropolitan University, Cardiff, CF5 2YB UK; 5 MRC Epidemiology Unit, University of Cambridge School of Clinical Medicine, Institute of Metabolic Science, Cambridge Biomedical Campus, Box 285, Cambridge, CB2 0QQ UK

**Keywords:** Physical activity, Peers, Girls, Intervention, School

## Abstract

**Background:**

Girls’ physical activity levels decline to a greater extent than boys as they enter adolescence. Role model interventions offer a potential solution to combat this public health issue. This study reports findings of a feasibility study of the CHARMING (CHoosing Active Role Models to INspire Girls) programme, a 6-week after-school primary school-based, community linked, role-model intervention.

**Methods:**

Between January 2021 and August 2022, a feasibility cluster randomised controlled trial (cRCT), with process and health economic evaluations was conducted in South Wales. Secondary schools were recruited, along with their adjoining primary schools to recruit Year 5 girls (aged 9–10 years). Role models were recruited from the surrounding school community (community role models) and from each secondary school (peer role models). A survey of self-reported outcome measures and accelerometers were administered at baseline and at 12 months. Following baseline, six primary schools were randomly allocated to intervention or control (usual practice) on a 2:1 basis. Post-intervention delivery, observations (*n* = 30), focus groups (*n* = 13) and interviews (*n* = 22) were conducted to explore study and intervention acceptability, feasibility and fidelity. Five pre-specified progression criteria included: implementation, attendance rates, and acceptability of the intervention, as well as completion of the primary outcome, including levels of completeness.

**Results:**

One hundred and fifty-six girls from six primary schools (four intervention and two control) were eligible to take part. Of these, 96 (62%) and 97 (62%) Year 5 girls took part in the survey and accelerometer measures respectively, with 78 (81%) and 77 (79%) participating in the 12-month follow-up. Findings indicate that it is feasible to collect health-related quality of life information from 9- to 10-year-olds using a digital self-report survey completed in schools. Despite the impacts of the COVID-19 pandemic, three of the five criteria (implementation, acceptability and completion of primary outcome) for progressing to a full-scale evaluation were met. Process evaluation data provide understandings of why two criteria (attendance and completeness of the primary outcome) were not met. Overall, data suggest that acceptability and feasibility of the intervention were high, and that the intervention was broadly delivered as intended. Alterations to the study measures and the intervention were suggested to increase intervention acceptability and feasibility, including recruitment and retention and extending the length of delivery in line with original intentions (12 weeks).

**Conclusions:**

Findings suggest the CHARMING intervention and cRCT design are likely to be acceptable and feasible, subject to further intervention and evaluation design optimisation.

**Trial registration:**

ClinicalTrials.gov ISRCTN36223327. Registered March 29, 2021.

**Supplementary Information:**

The online version contains supplementary material available at 10.1186/s40814-025-01615-7.

## Key messages regarding feasibility

- What uncertainties existed regarding the feasibility?
  We conducted a feasibility randomized controlled trial to determine if a school-based, community linked, role model physical activity intervention would be feasible and acceptable to inform a potential future large-scale study. As the future evaluation design required, a primary-secondary school dyad design, prior to the study uncertainties existed especially regarding recruitment, feasibility, and adherence.
- What are the key feasibility findings?
  CHARMING was feasible to deliver in primary schools, with peer role models sourced from adjoining secondary schools. CHARMING was acceptable to key stakeholders across schools and community settings. No adverse events were observed.
- What are the implications of the feasibility findings for the design of the main study?
  CHARMING is an acceptable and practical intervention which was deemed beneficial by the participants. Findings provide important information about study recruitment and retention, with avenues for further improvement identified. Further optimisation work for role model recruitment activities should be conducted, with an increased number of taster sessions delivered at schools.

## Background

Physical activity is a modifiable lifestyle factor that offers various potential health benefits [1, 2]; reducing chronic disease risk [3, 4], acting as a buffer against negative health behaviours [5, 6] and improving psychological wellbeing [7] and academic performance [8]. Many young people, however, do not meet current guidelines of 60 min daily moderate-to-vigorous physical activity (MVPA), with global accelerometery data revealing that only 29% of young people aged 2–18 years meet these guidelines [9]. A gradual age-related decline in MVPA has been shown from as young as 5 years, with estimated annual declines of 4% [10]. Recent self-report data in Wales, United Kingdom (UK), estimate that only 14% of 11–16-year-olds meet current physical activity guidelines of an average of 60 min of MVPA per day, with girls fairing less favourably than boys (10% vs. 18%) [11]. This gender disparity is consistently demonstrated among global estimates [10, 12, 13].

Considering the persistent gender gap in physical activity levels over the last decade, there are global calls for urgent action to address very low levels of physical activity among girls [14]. Promoting physical activity among girls contributes towards the 2030 Sustainable Development Goals through promoting policy level actions to empower women [15]. Current efforts are focused on older children, with UK-based programmes targeting adolescents or adults (i.e. ‘Girls Together’ in Wales [16], ‘US Girls’ across the UK [17], ‘This Girl Can’ in England [18]). Research demonstrates however, the need for greater emphasis on childhood, with the introduction of preventative methods at an earlier timepoint [19]. Promoting healthy behaviours and equitable gender norms specifically throughout preadolescence has been highlighted as a transformational opportunity to produce immediate and life course impacts [20, 21]. This period is important for fostering a positive association with physical activity, as early experiences with physical activity can lay the foundation for life-long engagement and support the development of habits that promote long-term health and well-being. The transition to adolescence is crucial to implement preventative strategies to help reduce the typically observed age-related decline in physical activity. One study involving 8–12-year-olds highlights the need to devise different types of intervention approaches for boys and girls, with differing mechanisms underlying physical activity levels and extracurricular physical activity notably playing a central role [22].

Despite the well-documented influences on girls’ physical activity levels, challenges in implementing gender-responsive physical activity interventions continue to persist while inequalities endure [23]. Reviews and meta-analyses have recently demonstrated larger effects among physical activity intervention designs which are ‘girls only’, schools-based, multi-component and underpinned by theory [24, 25]. Literature identifies the use of role models (‘someone who influences an individual through exemplar behaviours’ [26]) as a potential strategy to inspire young girls to become involved in, or maintain involvement in, physical activity and sport [27]. A longitudinal Australian study reported that adolescent girls were significantly more likely to be active if they had a role model [28] while the family-based DADDEE programme, involving dads as role models, showed positive impacts on girls’ physical activity levels [29]. This intervention approach has been internationally endorsed by the World Health Organisation (WHO) with specific recommendations for the use of role models within local communities to increase physical activity among females [22]. The National Institute for Health and Care Excellence (NICE) recommends that practitioners leading physical activity initiatives, including youth leaders, teachers, coaches and volunteers, should provide appropriate role models [1].

We designed a theory-informed, primary school-based role model intervention (CHARMING) using a participative community approach with children and stakeholders [30]. The intervention includes community role models (physical activity providers from local communities surrounding schools) and peer-role models (older girls from secondary schools). A purpose of the primary-secondary group design was to facilitate the links between schools and support pupil transition into secondary school. At present, interventions focusing on the transition phase are limited. The main aim of this study was to assess the feasibility and acceptability of CHARMING and its proposed evaluation methodology, to inform whether and how to proceed to a full-scale evaluation of the intervention.

## Objectives

The four objectives of the study were to:

1. Identify effective means of recruiting schools, participants and community and peer role models.
2. Assess the feasibility of conducting an effectiveness trial, health economic evaluation and assess the implementation of the intervention.
3. Explore the acceptability of the intervention and the influence of school context on intervention implementation.
4. Assess the extent to which each of five progression criteria (see Table 1) for conducting a full-scale trial are met.

**Table 1:** Progression Criterion, assessment methods and overview of results

Progression criteria		Assessment	High-level overview of results
**1**	**Is the intervention implemented with fidelity (in a manner in line with intervention theory) in at least 3 of 4 schools?**	Percentage of weekly physical activity taster sessions delivered (quantitative data)	Two schools delivered all 6 (100%) sessions and two schools delivered 5/6 (83.3%)
		Observation of whether sessions are delivered by community role models with peer role model (PRM) participation (quantitative data)	• 21/22 sessions were delivered by a CRM. One session was delivered by PRMs due to CRM illness • Dynamic relationships emerged between CRM’s & PRM’s • The observation log also noted that (after researcher adjustment) 83% of the time PRMs were participants in sessions and 44% of the time they led part of the session. 72% of PRMs interacted with the Year 5 girls and 27% shared information about the activities in their school
		Exploration of fidelity to the intervention delivery (quantitative and qualitative data)	• Observation data showed that sessions were 56 min in length (approximately 43 min dedicated to activity delivery) • Interview and focus group data indicated that all sessions were delivered in line with the one-hour taster session approach on school premises
		Observation and qualitative exploration of Q&A opportunities and signposting to community physical activity (quantitative and qualitative data)	• Observation forms showed pupils generally had good opportunities (85%) for questions. Limited interview and focus group data however suggest this did not take place frequently • Observation data indicated signposting within 17/24 sessions (71%). Verbal signposting was far more common (88%) than signposting via distribution of printed materials (41%). Qualitative data suggest signposting was inconsistent across schools and sessions, which was linked to the impact of COVID-19 and time
		Qualitative exploration with teachers, community and peer role models, and children regarding choice of activities delivered and engagement with role models (qualitative data)	• Observation data indicated all activities were deemed appropriate for the girls and choices were usually given within activities and viewed positively • Observation data showed that overall CRM’s used encouraging language, they were kind and sincere in their interactions with the pupils, and there was good rapport between the role models and the pupils across all sessions in all schools. Qualitative data indicated that session delivery was consistent with key principles of SDT such as competence and relatedness. Most CRM’s emphasised the importance of using positive, encouraging language, and building rapport with the girls as well as gradually improving their competence to perform certain skills. Data revealed less consistency relating to autonomy. While some CRMs described giving girls choices within structured plans and activities, some girls and peers identified wanting more choice and autonomy • Perceptions of intervention engagement varied among participant accounts and across school dyads. Value of former pupils was emphasised by teachers across all clusters in both primary and secondary schools. PRMs were perceived to be more relatable and engaged. Engagement described as improving over time by some as PRMs and girls had opportunities to build relationships. Evidence of consistency with principles of SDT, apart from autonomy
**2**	**Do at least 50% of consenting children in the intervention schools attend 50% of the scheduled sessions?**	The percentage of consenting children with recorded attendance at each scheduled intervention session per school (quantitative)	34.7% of assenting children in the intervention school attended 50% of the scheduled sessions (using imputed mean)
		Qualitative exploration with teachers and children regarding engagement with sessions (qualitative)	• **Attendance:** Fluctuating attendance levels across all schools with reported reasons: - Unclear communication of who was eligible to attend the intervention - Lack of childcare in one school - Two schools significantly impacted by COVID-19 - Difficulty of the first session put girls off in one school - Inherent challenges engaging with parents and families • **Timing:** After school seen as best/most feasible time to deliver the intervention by most participants although some did note that delivering in school time would promote greater reach as after school could be difficult for some girls’ families. Data highlighted some considerations for offering the intervention within school time • **Enjoyment:** Observation and qualitative data showed fairly consistent enjoyment scores across all session. This was linked to the opportunities to try new sports, be active, do sports that pupils liked, be with friends and health impacts. Enjoyment was also linked to the environment CRMs created and their approaches to delivery • **Engagement:** Observations data indicate that all pupils were fully engaged with all bar one session in which the majority of pupils were still engaged. Qualitative accounts supported these observations of engagement
**3**	**Does the process evaluation indicate the intervention is acceptable to children, parents, school staff and role models?**	Exploration of acceptability of intervention design and components (qualitative)	• **1-h** **taster PA sessions at Primary School:** Seen as convenient for the primary schools, primary school girls and CRMs. Many participants across all groups suggested the intervention should be delivered over a longer number of weeks, some also suggested having longer individual sessions or delivering more than one session a week. Data highlighted a need to consider transport options for future delivery and the option of delivery on secondary school premises (to further support pupil transition and provide an introduction to high school PA) • **Choice of activities:** Consistent feedback that from all participants that girls enjoyed having the chance to try different activities and it was activities that the schools did not offer or that it was activities the girls liked doing • **Community role models:** Valued by primary schools and primary schools girls, considering them knowledgeable, helpful, and supportive. Variation in the value and helpfulness of CRMs was discussed by PRMs and girls. Some CRMs would have liked more information on the schools attending, including numbers • **Peer role models:** Most described high levels of enjoyment of being involved in CHARMING and outlined different development opportunities that arose (e.g. learning new physical activity skills and techniques; opportunities to lead activities and try new physical activities, developing communication skills etc.). Some PRMs also liked being able to go back to their old primary schools. PRMs were often considered more relatable to young girls than CRMs • **School dyads:** Data highlighted the perceived value of linking primary and former secondary school pupils. Suggestions for improving future delivery included session delivery at secondary schools and opportunities for primary school girls to meet peers across other primary schools. PRM also suggested clearer communication and definition of roles and responsibilities for their future involvement
		Acceptability of single sex targeting among boys excluded from the intervention (qualitative)	• Among the two focus groups, most boys reported feeling that having a girls only intervention was important, recognising that there should be equity in opportunity. These views were echoed by teachers. Some boys reported that they would have liked to have taken part in an after-school PA programme and in the study measures. Some boys suggested that they think a programme involving competitive elements between boys and girls would be good
		Pupil and teacher perceptions of any inequalities in engagement with the intervention and if the intervention is likely to widen or reduce inequalities processes (qualitative)	• **Reach amongst preadolescent girls**: Intervention identified as an important opportunity for girls. Many girls described already being active, but some did discuss the intervention reaching those who would not usually be active/increasing their physical, some girls also discussed not usually being that active. Some teachers noted that delivery within school time would promote greater reach as after school could be difficult for some girls and families • **Displacement of other PA:** One teacher reported that CHARMING was delivered at the same time as another programme which girls initially preferred to go to. However, this desire reduced as week on week they were perceived to be more excited about CHARMING
**4a**	**Are at least 3 of 4 intervention schools and 1 of 2 control schools retained throughout the study?**	The percentage of primary schools retained throughout the study If > 1 school in either arm is not retained, the trial is unlikely to be feasible	All schools were retained throughout the study
**4b**	**Do at least 80% of children approached complete the baseline and follow-up accelerometer measures?**	Accelerometery data (% of children returning an accelerometer at baseline and at follow-up) Proceed: 80% Stop: < 50%; Review: 50–79%	100% returned at baseline 96.1% returned at follow-up General view that the study measures were feasible and acceptable. However, burden on school staff and amount of administrative work a significant challenge for all schools
**5**	**Do at least 70% of recruited children who receive an accelerometer return valid data (**≥ **3 days of 600 min including 1 weekend day) at baseline and follow-up for the primary outcome measure?**	Accelerometery data Proceed: 70% or more; Stop: < 40%; Review: 40–69%	Baseline: 58/91 (63.7%) Follow-up: 41/72 (56.9%)

*PC* Progression criterion, *PRMs* Peer role models, *CRM* Community role models, *PA* Physical activity, *PST* Primary school teacher (also showing participant ID number), *SLT* Senior lead teacher (also showing participant ID number), *SDT* Self-determination theory, Progression zone = proceed (green), review (amber) and stop (red)

While this paper provides findings on all four objectives, additional findings from objectives two and three will be provided within a health economics paper and separate qualitative paper [31].

## Methods

### Study design

This was a feasibility study including a two-armed cluster randomised controlled trial (cRCT), with process and feasibility health economic evaluation (ISCRTN 36223327), in six primary schools across South Wales between January 2021 and August 2022. For each school, publicly available data were collected on school size (number of pupils), percentage of children eligible for free school meals (proxy measure for socio-economic status) and pupil ethnicity.

### Sample size

The study aimed to provide estimates of key parameters for a future full-scale trial, rather than to power sufficiently to detect statistically significant differences [32]. We aimed to recruit 90 participants from six schools (average of 15 girls in Year 5 in six schools). This allowed for an estimation of feasibility criteria with reasonable precision across a diverse range of contexts (e.g. 70% returning valid primary outcome data estimated within ± 9.5%; 80% completing follow-up to within ± 8.3%.) and also took the cluster design effect (intra-class correlation (ICC) coefficient of 0.02) [33] into account.

### Sampling and participants

School recruitment took place between the start of March and end of April 2021. Six mainstream secondary schools across South Wales agreed to take part in the study. For each secondary school, all adjoining primary schools were invited to take part. The final sample included three groups, each comprising one secondary school and two adjoining primary schools. Within each of the six primary schools, all Year 5 girls (aged 9–10 years) were invited to participate in the study. Children who could not engage in physical activity due to medical reasons were excluded. To participate, Year 5 girls required parent opt-in consent in addition to providing their own assent.

### Data collection

Quantitative data were collected at baseline (Time 0: Spring term of Year 5, May 2021) and follow-up (Time 1: Spring term of Year 6, March–April 2022). Process evaluation data were collected throughout intervention delivery (Groups 1 and 2: May–July 2021 or Group 3: September–November 2021), immediately post-intervention and 3 months later, with data collection taking place in primary and secondary schools.

### Randomisation

Primary schools were randomly allocated following baseline data collection, at a 2:1 intervention:control ratio using a random number generator. Four were allocated to the intervention arm and two to the control arm by an independent member from the Centre for Trials Research who was blind to school identity. Schools were stratified by Local Authority (LA). The statisticians and all team members except the Principal Investigator and Trial Manager were blind to allocation. Group 3 contained two intervention schools.

### Intervention

The four intervention schools received a 6-week after-school intervention involving a 1-h weekly physical activity taster session, with a different activity delivered each week. The intervention was theory-informed, integrating self-determination theory (SDT) [34] and the socio-ecological model [35, 36]. At each school, teachers consulted with the girls before the intervention began to gather their preferences for the types of activities they would like to be delivered. These choices were then aligned with the available options from the current community provision. Sessions were delivered on the school premises by a different community role model each week and included an opportunity for questions and answers and signposting to community opportunities (i.e. highlighting times and days of sessions taking place within the local community). Each session also involved several peer role models (older girls from adjoining secondary schools) who participated alongside the primary school girls in each session. The peer role model component was a new addition to the earlier intervention design [30]. To support delivery of the intervention, the coordinating teacher in each primary and secondary school as well as the community and peer role models were provided with intervention manuals and guidance. All sessions took place in-person. The intervention and its implementation are described in greater detail within the protocol [37].

### Controls

Control schools were asked to continue with their usual physical activities during the period of the intervention but were provided with intervention materials (e.g., list of community role model contacts and organisations) at the end of the study.

### Outcome measures

The key outcome of the study (objective 4) was whether pre-specified progression criteria (PC) were met to progress to a full-scale trial. Table 1 displays each progression criterion and how they assessed feasibility of the intervention and study measures.

The primary outcome assessed for use in a future trial was average daily minutes spent in MVPA. Participants were asked to wear a GT3X ActiGraph accelerometer on the right hip for seven consecutive days (during waking hours) and complete a monitor wear diary. The diary asked participants to keep a record of the time they removed the belt, reason for removal and time they placed the belt back on. This allowed any water-based activities such as swimming to be captured.

Participants were asked to complete an online survey which collected data on demographics, self-reported physical activity [38], psychosocial outcomes, current after-school sport or physical activity engagement and health-related quality of life [39, 40].

All participants across the six primary schools were invited to participate in the accelerometery and online survey at baseline and follow up. Full details of the survey outcomes are available within the protocol [37].

### Adverse events

A safeguarding protocol was developed for the study and teachers were provided with an Incident Template Form prior to intervention delivery. All schools also adhered to a COVID-19 risk assessment. Process evaluation methods explored unintended consequences and potential harms. The study was deemed as low risk to participants.

### Process evaluation

Data collection was carried out in secondary and primary schools participating in the intervention (i.e. not in control schools). Focus groups were conducted with primary school pupils (separate groups of boys and girls) and peer role models (1 per secondary school). As this was a girls-only intervention, the focus groups with boys aimed to explore acceptability and potential unintended consequences of a targeted intervention for girls. The primary school girls’ focus groups took place at two timepoints; immediately after the intervention and 3 months post intervention. The focus groups with boys and peer role models took place immediately after the intervention concluded. One-to-one semi-structured interviews were conducted post-intervention with; two primary school contacts (school senior management member and the lead teacher involved in overseeing the intervention); parents of participating children (approximately 2 per school), the secondary school lead teacher and community role models (approximately 4 per school). Session registers and observation forms were completed by the primary school lead teacher for each intervention session (*N* = 6 per school). A member of the research team also observed two random sessions per school (*N* = 8) as a form of verification of teacher observation records. A total of 83 participants took part in either an interview (*n* = 22) or focus group (*n* = 13) as part of the process evaluation. Table 2 shows the sample and aims for each data collection method. Additional file 1 displays the questions asked throughout interviews and focus groups.

**Table 2:** Overview of data collection methods, timings and aims

Method	Participant (age range; job role if applicable)	Sample (*N*)	Conduct (*N*)	Aims of data collection
Focus group	Girls (aged 9–11* years) × 2	18	V (1) F (3)	Post-intervention: Explore experiences of the intervention, acceptability and barriers and facilitators to participation (RQ6,9). Examine factors that might have affected recruitment and attendance (i.e. reach), delivery, enjoyment, and anything that could be improved (RQ3)
	Girls (Aged 9–11 years) × 2	13	V (1) F (3)	3-month follow-up: Explore experiences of the intervention, acceptability and barriers and facilitators to participation (RQ6,9). Examine factors that might have affected recruitment and attendance (i.e. reach), delivery, enjoyment, and anything that could be improved (RQ3)
	Boys (aged 9–10 years)	12	F (2)	Explore the acceptability and potential unintended consequences of a targeted intervention for girls (RQ8)
	Peer role models (aged 12–16 years)	18	V (1) F (2)	Explore acceptability of the intervention and facilitators and barriers to recruitment of role models (RQ2,10). Examined factors that might have affected recruitment and attendance (i.e. reach), delivery, enjoyment, and anything that could be improved (RQ3)
Interview	Primary School Senior Leadership Team (Headteacher *n* = 3)	3	V (3)	Explore acceptability and feasibility of intervention; (RQ6,9). Examined factors that might have affected recruitment and attendance (i.e. reach), delivery, enjoyment, and anything that could be improved (RQ3)
	Primary School Lead (Teacher *n* = 2 School’s 1 & 3; Teaching Assistant *n* = 2 School’s 5 and 6)	4	V (4)	Explore intervention acceptability and feasibility of implementation; RQ6,9). Examined factors that might have affected recruitment and attendance (i.e. reach), delivery, enjoyment, and anything that could be improved (RQ3)
	Parents of participating Primary School Girls	2	V (2)	Parents of participating children (2 per intervention school; RQ9) at 14 weeks to allow reflection on the processes of implementing the intervention and to explore overall feasibility and impact of the intervention. Examined factors that might have affected recruitment and attendance (i.e. reach), delivery, enjoyment, and anything that could be improved (RQ3)
	Secondary School Lead (Cluster 1 and 2—Assistant Head Teacher *n* = 2; Cluster 3—PE Teacher *n* = 1)	3	V (3)	Explore intervention acceptability and feasibility of implementation (RQ6,9). Examined factors that might have affected recruitment and attendance (i.e. reach), delivery, enjoyment, and anything that could be improved (RQ3)
	Community role models	10	V (10)	Explore acceptability of the intervention and facilitators and barriers to recruitment of role models (RQ2,10). Examined factors that might have affected recruitment and attendance (i.e. reach), delivery, enjoyment, and anything that could be improved (RQ3)
Session registers	Teachers	22 forms	F (21)	Record those present at each session of the intervention on a session register, noting those absent from school on that day and any children who opted out of taking part in the intervention. PIDs used to track each pupil’s attendance across each session
Observation forms	Teachers and Researchers	30 forms	F (29)	Record whether community role models delivered the planned core components of each session fully, partially or not at all

Data collection took place in Summer 2021 (Cluster 1 and 2 = Schools 1 and 2) or Autumn 2021 (Cluster 3 = Schools 5 and 6)

*N* Number, *F* Face-to-face, *V* Virtual, *RQ* Research question, *N*/*A* Not applicable, *PE* Physical education, *PID* Participant identification number

### Health economics evaluation

Health-related quality of life (HRQoL) and intervention cost data were collected and analysed to test the feasibility of conducting a health economic evaluation in a future full-scale trial. The feasibility of using the Child Health Utility 9D (CHU-9D) [39] and the EuroQol 5D Youth (EQ-5D-Y) [40] measures as a means of assessing children’s HRQoL was explored. Students from three schools completed the EQ-5D-Y, and in the other three schools, students completed the CHU-9D to reduce burden on pupils completing the questionnaires, whilst retaining the ability to answer feasibility questions of using each measure in a future effectiveness evaluation. Resource use data extracted from the teacher, parent and community role model interviews examined the feasibility of identifying and measuring the resources required to deliver the intervention.

### Statistical analyses

Descriptive statistics (frequencies, percentages, means, standard deviations) were used to describe participant recruitment, attendance and observation data. Summaries of intervention reach and fidelity were presented over time, overall and by school. Accelerometry data were processed using ActiLife 6 software and analysed using a batch processing protocol. Non-wear time was determined by continuous periods of 60 min of zero counts. Valid wear time criteria were a minimum of 3 days (i.e. 600 min) including 1 weekend day. Applying Evenson cut-points [41], the average daily minutes spent sedentary, in MVPA and time-segment-specific time spent in each activity intensity (e.g. at weekends) were estimated and summarised overall and by trial arm. Further information is provided in the study protocol [37].

A two-level hierarchical mixed-effects linear regression model (with participants nested within schools) was used to estimate direction of intervention effects on the pilot primary outcome of accelerometer-measured physical activity, and the adjusted mean difference reported (intervention and control), alongside a 95% confidence interval (CI). The school-level ICC coefficient for average daily minutes of MVPA over the 7 days was estimated alongside a 95% CI. Continuous secondary outcomes were analysed using the same method. For binary outcomes (participation in school/non-school clubs or not), a logistic regression model was used and parameter estimates reported as odds ratios (alongside a 95% CI).

All analyses used a modified intention to treat approach (i.e. students were analysed in the groups in which their school was randomised to, regardless of adherence to the intervention) and missing outcome data were not replaced except for attendance data. Statistical analyses were conducted using Stata 16 software [42]. The analysis and reporting of this feasibility cRCT are in accordance with CONSORT (Consolidated Standards of Reporting Trials) guidelines [43, 44] (see Additional file 2).

### Qualitative analyses

Interviews and focus groups were audio-recorded and transcribed verbatim and fully anonymised prior to analysis. NVivo v12 (QSR International Pty Ltd) software was used to manage and analyse data. Using thematic analysis (combining both deductive and inductive elements), all focus group and interview data were analysed to examine acceptability, feasibility, fidelity and engagement. The coding framework was discussed between the study team to finalise the themes and sub-themes. Triangulation of the process evaluation data was used to combine qualitative and quantitative data analyses. Themes were summarised at a higher level as part of the triangulation and will be reported within a separate qualitative paper.

### Health economic analyses

Response rates for the EQ-5D-Y and CHU-9D at baseline and 12-month follow-up were explored using descriptive statistics. Ceiling effects in both HRQoL measures were investigated by assessing the proportion of participants that state ‘no problems’ across all dimensions of the measures. Micro-costing cost estimation methodology [45], from a local authority (LA) perspective, was applied to information extracted from qualitative data to explore if it was feasible to identify and measure the resources required to deliver the intervention to estimate a cost per programme and per participant. Full details of the health economic analyses undertaken and results will be published in a separate health economics paper.

## Results

### Objective 1—effective means of recruitment

#### School recruitment

In total, 35 secondary schools were invited to participate in the study. Participation involved the recruitment and involvement of secondary school pupils as peer role models within intervention delivery. The six secondary schools agreeing to participate (17.14%) were located within two LAs, one city-based and one in a town region. Both LAs contained a high number of deprived areas, between 45 and 60% of Lower Super Output Areas within each LA were classified as in the top 50% most deprived ranks in Wales. For each secondary school, all feeder primary schools (*N* = 37 in total) were invited to participate, eight expressed an interest, 24 did not respond, five declined and six were subsequently enrolled into the study.

The COVID-19 pandemic posed significant challenges to the recruitment of both secondary and primary schools. Several secondary schools were unable to take part due to the inability of mixing student groups (i.e. class bubbles) and numerous primary schools reported that no extra-curricular activities were allowed to run at the school due to safety procedures. As a result, we were limited in our ability to recruit both primary and secondary schools where their adjoining schools were unable to participate. The final study sample involved three secondary schools, each with two adjoining primary schools.

Among the six recruited primary schools, the average school size was 423 pupils (range 123–695), with a mean percentage of pupils eligible for free school meals at 24.5% (range 4.4–55.6%) and proportions of Black, Asian, and Minority Ethnic pupils ranging between 9.8–30.3%.

#### Pupil recruitment

**Fig. 1:**
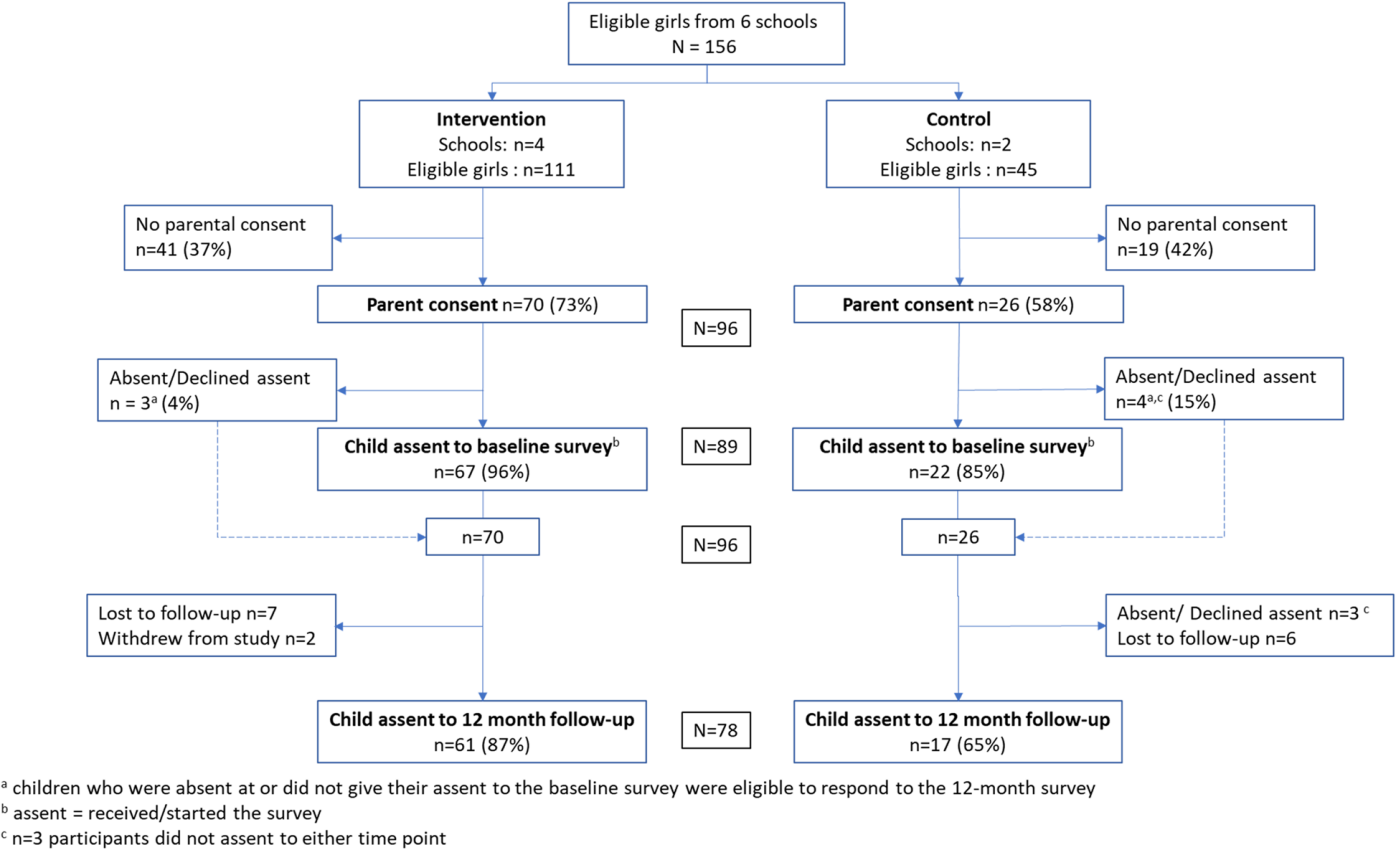
CONSORT diagram for survey for participants and schools by trial arm

**Fig. 2:**
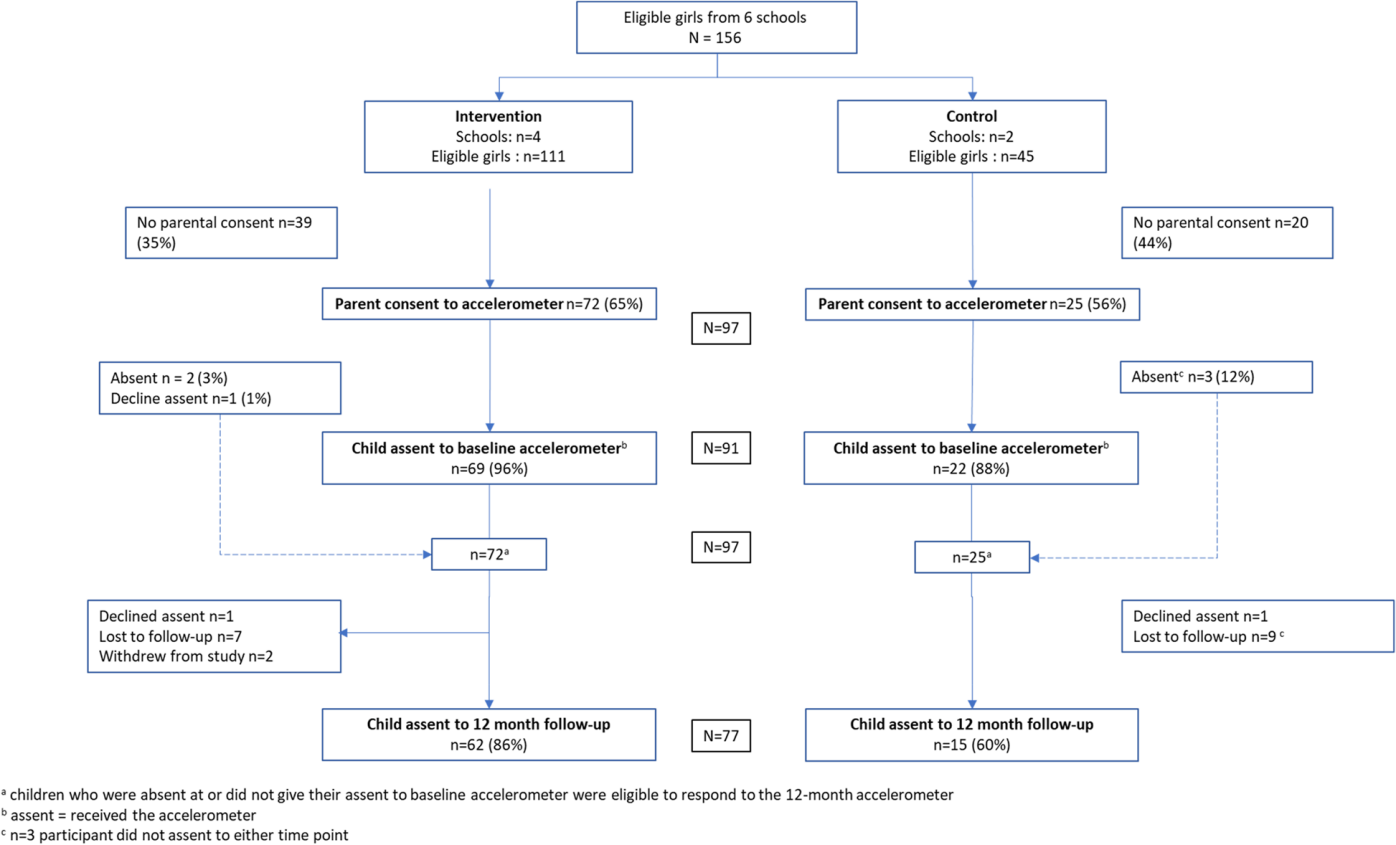
CONSORT diagram for the accelerometer for participants and schools by trial arm

Across the six primary schools, 156 Year 5 girls were eligible to take part in the study. Participant recruitment and retention in the survey and accelerometery are shown in Figs. 1 and 2 respectively. Of those eligible, parental consent was received for 96 girls (62%) for survey participation and 97 girls (62%) for the accelerometer measure. From these, a total of 95 girls (Intervention: 72 vs. Control: 23) completed a survey or accelerometer at either time point. Descriptive statistics for study participants are shown in Table 3. Participant characteristics were broadly comparable across intervention and control schools.

**Table 3:** Demographic characteristics and descriptives of assenting children (either survey and/or accelerometer) by trial arm

	**Intervention**		**Control**	
	**% / M (SD)**	***N***	**% / M (SD)**	***N***
Parental consent to accelerometery	*n* = 72		*n* = 25	
Parental consent to survey	*n* = 70		*n* = 26	
**Completed survey or accelerometer at either baseline or follow-up**	*n* = **72**		*n* = **23**^a^	
Participants per school setting				
1	37.5	27	–	–
2	–	–	69.6	16
3	27.8	20	–	–
4	–	–	30.4	7
5	26.4	19	–	–
6	8.3	6	–	–
**Sociodemographic information**				
Age in years	10.2 (0.3)	62	10.3 (0.3)	20
* Not recorded*		*10*		*3*
Ethnicity—Black/Asian/Mixed Race/Other	18.9	53	35.3	17
* Not recorded*		*19*		*6*
**Measures – all reported as M (SD)**				
FAS scale [Score range 0 = less to 10 more affluent]	7.2 (1.8)	54	6.9 (1.7)	15
*Not recorded*		*18*		*8*
PAQ-C scale [Score range 1 = less to 5 = more physical activity]	3.0 (0.7)	44	3.1 (0.6)	21
*Not recorded*		*28*		*2*
LCQ Autonomy scale [Score range 6 = less to 36 more autonomy]	25.9 (6.4)	48	24.7 (7.1)	17
*Not recorded*		*24*		*6*
LCQ Competence scale [Score range 6 = less to 36 more competence]	23.7 (6.1)	47	24.4 (6.3)	16
*Not recorded*		*25*		*7*
LCQ Relatedness scale [Score range 6 = less to 36 more relatedness]	24.6 (7.4)	47	24.7 (6.7)	17
*Not recorded*		*25*		*6*
**Participation in school clubs**				
School sport club/PA at least 3/week- *n*(%) Yes	10.5	57	9.5	21
* Not recorded*		*15*		*2*
Non-school sport club/PA at least 3/week- *n*(%) Yes	27.9	61	35.0	20
* Not recorded*		*11*		*3*
**Accelerometer data**		72		25
Did not receive an accelerometer (at baseline)	4.2	3	0	0
Parental consent but no child assent		0	0	3
Received an accelerometer		69		22
**Non-valid**	*n* = **22**		*n* = **11**	
No readings	1.4	1	4.5	1
Less than 3 days data	11.6	8	22.7	5
≥ 3 days data but no weekend	18.8	13	22.7	5
**Valid**	*n* = **47**		*n* = **11**	
≥ 3 days with weekend	68.1	47	50.0	11
Daily minutes spent in MVPA M (SD)	48.9 (15.7)	47	46.6 (18.3)	11
Daily minutes spent sedentary M (SD)	518.1 (68.0)	47	508.2 (65.4)	11

All results are presented as *n* (%) unless otherwise specified. *M* mean, *SD* standard deviation, *FAM* Family affluence measure, *PAQ*-*C* self-reported physical activity, *PA* Physical activity, *LCQ* Learning Climate Questionnaire, *MVPA* Moderate to vigorous physical activity

^a^*n* = 3 in control arm did not complete a survey at either time point

Two participants withdrew after baseline data collection. Of the 96 participants, 78 (81%) completed the 12-month follow-up survey, with a higher proportion of girls from the intervention schools compared to control schools (87% vs. 65% respectively). Similarly, with the accelerometery measure, 77 (79%) were followed up at 12 months (Intervention: 62/72 (86%) vs. Control: 15/25 (60%)). Participant characteristics are shown in Table 4.

**Table 4:** Baseline demographics and results (participants assenting to baseline survey, completing follow-up, or lost to follow-up)

	**Parental consent**	**Baseline**		**12-month follow-up**			
		**Child assent**^**a**^		**Child assent**^**a**^		**Declined/no assent**	
	***N***	**%**	***N***	**%**	**N**	**%**	***N***
**Trial arm**	96		89		78		18
Intervention	70	95.7	67	87.1	61	12.9	9
Control	26	84.6	22	65.4	17	34.6	9
Number of participants per school setting					78		18
1	27	29.2	26	26.9	21	33.3	6
2	19	18.0	16	16.7	13	33.3	6
3	20	21.4	19	25.6	20	–	–
4	7	6.7	6	5.1	4	16.7	3
5	17	18.0	16	18.0	14	16.7	3
6	6	6.7	6	7.7	6	–	–
**Sociodemographic information**							
Age in years M (SD)	–	10.2 (0.3)	82	10.2 (0.3)	70	10.2 (0.3)	12
* Missing*			7		8		6
Ethnicity			77		65		12
White	–	70.1	54	70.8	46	66.7	8
Black/Asian/Mixed Race/Other	–	20.8	16	20.0	13	25.0	3
*Do not want to answer*		9.0	7	9.2	6	8.3	1
FAM scale [Score range 0 = less to 10 more affluent] M (SD)	–	7.1 (1.8)	69	7.2 (1.9)	57	6.5 (1.2)	12
**Measures—all reported as M (SD)**							
PAQ-C scale [Score range 1 = less to 5 = more physical activity]	–	3.0 (0.7)	65	3.1 (0.6)	54	2.8 (0.7)	11
LCQ) Autonomy scale [Score range 6 = less to 36 more autonomy]	–	25.6 (6.5)	65	25.3 (6.6)	54	26.8 (6.6)	11
LCQ Competence scale [Score range 6 = less to 36 more competence]	–	23.9 (6.1)	63	23.7 (6.0)	54	24.6 (7.1)	9
LCQ Relatedness scale [Score range 6 = less to 36 more relatedness]	–	24.6 (7.1)	64	24.6 (7.2)	55	24.9 (7.2)	9
**Participation in sport clubs**							
School sport club/PA at least 3/week			78		65		13
Yes	–	10.3	8	9.2	6	15.4	2
No	–	89.7	70	90.8	59	84.6	11
Non-school sport club/PA at least 3/week			81		68		13
Yes	–	29.6	24	29.4	20	30.8	4
No	–	70.4	57	70.6	48	69.2	9
**Accelerometer data M (SD)**							
Daily minutes spent in MVPA^b^	–	48.5 (16.2)	57	49.3 (16.9)	49	43.6 (10.5)	8
Daily minutes spent sedentary^b^	–	516.1 (68.3)	57	512.5 (69.7)	49	538.1 (58.3)	8
**Intervention Attendance**							
Sessions attended (imputed score)^c^ Median (25th to 75th centile)	–	2.4 (0 to 4.8)	67	2.7 (0 to 4.9)	61	0 (0 to 4.8)	9
Sessions attended (non-imputed score)^c^ Median (25th to 75th centile)	–	2 (0 to 4)	67	2 (0 to 4)	61	0 (0 to 4)	9
No participation in any sessions^c^	–	29.9	67	27.9	58	66.7	9
Attended at least 50% of sessions	–	37.3	67	36.1	61	33.3	9

All results are presented as *n* (%) unless otherwise specified. *M* mean, *SD* standard deviation, *FAM* family affluence measure, *PAQ*-*C* self-reported physical activity, *PA* physical activity, *LCQ* Learning Climate Questionnaire, *MVPA* moderate to vigorous physical activity

^a^Assent = received/started the survey

^b^Assented to accelerometer at baseline and had valid data *n* = 91

^c^Intervention group at baseline only (*n* = 67)

#### Role model recruitment

In total, 16 female community role models were recruited across the two LAs. Recruitment was largely coordinated by the study team with the support of community sports development teams and wider contacts, with minimal ownership by schools. This process was necessary due to challenges of the COVID-19 pandemic. Community role models’ day-to-day roles included; sports coach, club coach, activity instructor, business owner, university student and volunteer.

In total, 23 peer role models were recruited across the three secondary schools. Girls were typically recruited from Year groups 8–11 (ages 12–16-years) and each school adopted their own recruitment approach. In interviews with secondary school teachers, recruitment processes were described as simple and time efficient, with approaches largely targeted due to the time constraints and wider challenges of the COVID-19 pandemic. Teachers described peer role models as highly engaged students willing to do extra-curricular activities, and as strong performers in sport and school generally. In the future, teachers noted a desire to adopt a more inclusive recruitment strategy while student recruitment materials and transport logistics were areas identified for future improvement.

### Objective 2—feasibility of conducting an effectiveness trial and health economic evaluation

#### Consent

All six schools were retained throughout the study. Qualitative data (see Table 5 for exemplar quotes) from school staff broadly indicated that study opt-in consent procedures were a feasible method for collating consent, with this approach mirroring existing school practices for extra and non-curricular activities. Both parents also agreed that the consent process was acceptable. That said, a few teachers stated that an opt-out process would be preferable.

**Table 5:** Exemplar quotes to support qualitative findings

	**Exemplar quotes**
Objective 2—Feasibility of trial	
Consent	• ‘… for things like school trips or yes, anything like that, usually it’s an opt in, so, that was in line with what we do … especially because we were asking the girls to wear a monitor and keep track of that, it was an investment for them, an investment of time, so, I think it was fair that it, it was right that it was an opt in’ 1PST1 • ‘I think, I’ll be honest with you, when you address the parents, all the parents want to really know about is the actual activity (intervention)’ 5PST • ‘We normally, I’ve learnt this over time, we send letters out, or via schools comms and electronically, digitally, giving the information and then, obviously, on the bottom of the email, or whatever, comms are, if you don’t want your child to take part in this study, please contact, and even email.… Because otherwise, you’ve got to have somebody to collect the list of names, somebody chasing the names, oh no, too much time’ SSS01 • ‘… it’s never a high percentage of … bringing paperwork back in, is a no-go, if that makes sense. It has literally got to be, if you’ve got lots, if it’s a full A4 or two A4’s it’s very rare one of our parents would read that… Unless it’s not, you know, the major important ones, you do push it (*letter*) to one side, unfortunately’ 5PST
*Study Documents and School-Level Administration*	• ‘So, I really liked the sheet that you gave us which was for the girls, which was phrased, I thought it was phrased really well for their age, it was completely appropriate and they understood it really well … … it was really helpful that you < researcher > joined in with us for the session, when the girls were filling in the forms and giving out the activity monitors. I don’t think I would have been able to answer their questions in the way that you were able to, and to explain the project.’ 1PST1 • ‘Initially there seemed to be a lot of paperwork and there was a bit … I think we all had a bit of a panic and what have we signed up for? But I think the CHARMING project were really good with us, in changing that, and how it was set up, we were really happy with the way that it way that it was changed too. For us, the less admin side of it’ 6SMT3
*Study measures*	• ‘I think we could make it (*survey*) a little bit shorter, because it did take a while’ 3GFG • ‘It (*activity monitor*) was fun, they were OK to wear’ 6GFG • ‘But in school they all seemed happy to wear them and it didn’t affect their activity, it didn’t seem to bother them during the day at all, it was just a bit of a novelty at the beginning and then they seemed to get used to them’ 1PST1
Peer role model recruitment	• ‘… I probably would have encouraged other pupils that don’t necessarily do much in the community, to be part of this project, because some children do a lot of different activities, be it sport and drama and music and. So, I think it would have been an opportunity to tap into those pupils, maybe that are not part of everything. However, like I said, you probably noticed with the girls that you’ve got there, they are some of my best pupils and my best pupils in sport’ SSS01 • ‘… rather than giving the (recruitment) sheets initially, it was just having a chat, so this is what’s expected, do you like the idea of being leaders and, obviously, like I said, we did have certain girls in mind but we wanted to put to everyone and it just so happened, the girls we thought were going to go for it, straight away went for it really, so it was quite an easy process for us actually, trying to find these girls’ SSS03
Objective 3— Acceptability of intervention	
Aligning with school ethos	• ‘Especially, when you said about physical activity, I just feel like, that it is a blip there for this particular age, and I actually know what, that’s why, you know, I’ve noticed that girls are quite, they do give up quite easy. There’s that stigma, that it’s going to. If you understand what I mean, it’s like I find they’re too embarrassed to even attempt to do anything. That goes with the transition of high school as well. It’s that fear’ 6SMT • ‘I think you could really plug that mental health as well, because the mental health now is a national priority, and we’ve got the framework being released now in the next couple of weeks, months on the Whole School approach to mental health. Which is going to have to include physical activity’ 1SLT1
Enjoyment	• ‘I think the best thing about it, is that they’ve been able to take part in things that we don’t usually do at school. We tend to stick to activities where we have the equipment and we have the knowledge, in schools, so, we do the same sort of sports, every year. So, the chance to do gymnastics or basketball, or completely different activities, was, they really enjoyed that and a lot of them want to have the information, which was given at the end of the session to join after school clubs’ 1PST • ‘… And it was quite nice, because they weren't just super sporty ones, they were ones who don't do sport, you know. So, it was quite nice that … really unfit children don't ever do any exercise or sport, and they came every week, so I thought that was a really positive thing’ 6SMT3 • ‘What I learned was how to play squash because I never knew how to play it. So that was quite fun’ 6GFG
Girls only	• ‘Well I, because some girls like don’t want to do much sport or like they choose to, but I think because on TV mostly, if we look at Sports Channels, most of them are boys playing. So, they don’t have as much inspire …’ 3BFG • ‘It was actually really nice, just being girls, because we’re so used to having boys around us, as we do… But it’s nice just to be around people like the same as you, not the same looking as you, but knowing that they’re the same gender. And it’s, it’s nice to be just girls for once, instead of all the boys messing about and stuff’ 3GFG • ‘But … you know, the vision is here to get more girls participating, so you have to take something away (*boys*) for that to happen’ 1SLT1 • ‘Especially, because I thought it was quite nice, just the fact it was all girls, there’s no worries of doing something wrong in front of the boys’ 6SMT
Peer role models	• ‘Again, I thought it was really lovely that some of them were ex pupils, so, that was really nice and we could say to the girls “Oh I remember when such and such was in my class”, and funny little anecdotes … it’s them in a few years’ time isn’t it, and the fact that they were wearing their sporty things, and all joining in and then when we had a little chat, they go to netball and they go to gymnastics and they attend hockey and, so, it was really lovely peer role models. They were proper role models for them … and the fact that they were from [CHARMING Secondary School], which is one of our feeder schools, was great. They got to see [CHARMING Secondary School Teacher] and one of the other teachers, that will, potentially, be teaching them’ 3PST2 • ‘So, actually, that I think was a really vital part of the whole programme, … The girls or volunteers, being the age that they are, they’re also at the point where they’ve just made it through that usual drop out age of around ten, eleven, twelve. They’ve made it just past that sort of thing. So, it’s nice for them to be like oh, they’re still in the sport, whereas I’m just coaching it, they’re like, they’re actually still doing sports and that. So, even just that, and them just talking about what they do around that, is really good for them’ CRM5 • ‘One of these Year 9 s was really kind and they didn’t show off… but they showed me. She made me feel a bit confident, because after seeing her do it, it made me feel a bit more confident in myself. I’d seen all the Year 9’s do it and all my friends do it’ 3GFG • ‘They could have said, right we have to do this, keep going, keep going and keep coaching and encouraging us but no, they kept doing giggling … … I think they were embarrassed’ 3GFG3M
Community role models	• ‘I thought that all the coaches were fab’ 5PST • ‘I think a lot of them responded really well. One of the girls at the end of the session they were like, do we have you next Monday and I was like, oh no. She was like, no. So, I think that they like warmed to me quite well in the only hour that I had’ CRM18 • ‘I thought that everyone who’s come in, has been absolutely brilliant, the coaches, that’s what’s been the best thing, I think, about the programme, they’ve all been very knowledgeable, really engaging, really good with the girls’ 1PST1 • ‘I think, having the community peer role model, it’s that chance of assuming their coaches in maybe clubs in the community … it’s having those links for the kids to know where they can go. So, I think that’s something that has a lot of room for, like, positively, because I do think, a lot of our girls, they come, they do Physical Education, they might enjoy this sport or that sport and, a lot of the time they’re, like, well, what can I do with it? Where can I go in the community? And, like, a lot of the time, like, things aren’t that well-advertised or well known’ SSS03

*GFG* girls focus group, *CRM* community role model, *PST* primary school teacher, *GFG3M* girls focus group 3-months, *SMT* senior management team, *SSS* secondary school staff, *SLT* senior lead teacher

#### Study documents and school-level administration

Interviews with school staff indicated that study documents were clear and informative and were appreciative of the extra support and communication from the research team throughout the study. All primary schools however highlighted the high-level of work required in coordinating pupil recruitment and data collection sessions. Two intervention schools noted these processes as manageable, yet the other two intervention schools considered withdrawing from the study following baseline data collection due to staff workload concerns amid pandemic pressures.

#### Study measures

Regarding the conduct of study measures, the survey was generally viewed as positive by school staff and girls, alongside feedback to shorten the design and review question structure. Primary school girls were happy to wear activity monitors, with some reflecting that monitor wear encouraged them to be more active and others noting their parents encouraged them to be more active. However, some did report finding them uncomfortable to wear.

#### Accelerometery data

Among the 97 participants who had parent consent to receive an accelerometer, 6 (6.2%) participants were not present at baseline to receive it (Table 6). Of the remaining 91 participants who received an accelerometer, 2 (2.2%) had no valid data indicating that they did not wear the device at all (zero acceleration over the whole 7-day period), 13 (14.3%) accumulated some wear time but had less than three days data, 18 (19.8%) had 3 or more valid days but no weekend data and 58 (63.7%) had 3 or more valid days with weekend data. By trial arm, a higher percentage of participants in intervention schools met the 3-day accelerometer wear-time criterion at baseline than participants in control schools (68.1% vs. 50.0% respectively). Seventy-seven participants were retained to 12-month follow-up, of whom 41 (53.3%) provided valid useable accelerometery data, a reduction of 10.4% overall (11.6% in intervention schools and 10.0% in control schools). A total of 16 (19.5%) participants were absent at follow-up.

**Table 6:** Valid and non-valid accelerometer data at baseline and follow-up

Baseline					
	**Participants with baseline accelerometer (***n* = **97)**	**Intervention** **(***n* = **72)**	**Control** **(***n* = **25)**		
**Did not receive an accelerometer (not at baseline)**	3 (3.1%)	3 (4.2%)			
**Parental consent but no child assent**	3 (3.1%)		3 (12.0%)		
**Received an accelerometer**	*n* = **91**	*n* = **69**	*n* = **22**		
**Non-valid**					
**No readings**	2 (2.2%)	1 (1.4%)	1 (4.5%)		
**Less than 3** days** data**	13 (14.3%)	8 (11.6%)	5 (22.7%)		
** ≥ 3** days** data but no weekend**	18 (19.8%)	13 (18.8%)	5 (22.7%)		
**Valid**					
** ≥ 3** days** with weekend**	58 (63.7%)	47 (68.1%)	11 (50.0%)		
**Follow-up**					
	**All** *n* = **97**	**Participants present at baseline** *n* = **91**	**Received accelerometer at follow-up** **(***n* = **77)**	**Intervention: Received accelerometer at follow-up** **(***n* = **62)**	**Control:** **Received accelerometer at follow-up** **(***n* = **15)**
**No assent**	5 (5.2%)	1 (1.1%)	–	–	–
**Not at follow-up**	15 (15.5%)	15 (16.5%)	–	–	–
**Received an accelerometer**			**77**	**62**	**15**
**No data file**	2 (2.1%)	2 (2.2%)	2 (2.6%)	0	2 (13.3%)
**Did not return accelerometer**	3 (3.1%)	3 (3.3%)	3 (3.9%)	3 (4.8%)	0
**Non-valid data**					
** No readings**	18 (18.6%)	16 (17.6%)	18 (23.4%)	15 (24.2%)	3 (20.0%)
** Less than 3 days**	0	0	0	0	0
**≥ 3** days** data but no weekend**	13 (13.4%)	13 (14.3%)	13 (16.9%)	9 (14.5%)	4 (26.7%)
**Valid**					
**≥ 3** days** with weekend**	41 (42.3%)	41 (45.1%)	41 (53.3%)	35 (56.5%)	6 (40.0%)

Table 7 summarises the primary and secondary outcome variables at baseline and follow-up. In total, 30 participants provided valid accelerometer data at baseline and follow-up, from 5 schools (one control school did not have any valid data at both time points). The analysis of average daily minutes spent in MVPA showed an adjusted mean difference in change of 19 min between arms (95% CI: 1.35 to 36.70 min). Hence, while the study was not powered to estimate effects, the result is in the hoped-for direction with no evidence of harm. There was evidence of clustering of participants within schools (ICC = 0.01). This ICC should be interpreted with caution as the CI was not estimable due to a low number of participants, and likely to be wide. For the secondary outcomes, self-reported physical activity (Physical Activity Questionnaire for Older Children (PAQ-C)) [38], and sports club participation in and outside of school, at least three times a week, after adjusting for baseline measures, the point estimate favoured the intervention arm. There was a small degree of clustering with participants in school clubs than non-school clubs (ICC = 0.018 and 0.006 respectively). For all three subscales of the SDT questionnaire (autonomy, competence and relatedness) [34], after adjusting for baseline, the point estimate favoured the control group. Additionally, the average daily minutes spent sedentary was higher in the control group with an adjusted mean difference of 87 min and high indication of clustering (ICC = 0.291, 95% CI: 0.004 to 0.980).

**Table 7:** Primary and secondary outcomes at baseline and follow-up for the intervention group and a control group

	**Baseline**				**12 months follow-up**						
	**Intervention (***n* = **72)**		**Control (***n* = **23)**		**Intervention** **(***n* = **61)**		**Control** **(***n* = **17)**		**Adjusted mean difference**^**a**^ **(95% CI)**	**ICC (95% CI)**^b^	***n***
	**% / M (SD)**	***N***	**% / M (SD)**	***n***	**% / M (SD)**	***n***	**% / M (SD)**	***N***			
**Primary outcome:** Average daily minutes spent in MVPA^c^	48.9 (15.7)	47	46.6 (18.3)	11	64.4 (24.5)	35	44.5 (21.1)	6			
*Missing*		*25*		*12*		*26*		*11*			
Complete case analysis (baseline and follow-up data)	50.8 (19.8)	24	49.6 (23.0)	6	64.4 (25.5)	24	44.5 (21.1)	6	19.0 (1.35 to 36.70)	0.014	30^d^
**Secondary outcomes**											
PAQ-C scale^e^	3.0 (0.7)	44	3.1 (0.6)	21	2.9 (0.8)	52	2.8 (0.5)	14	0.19 (−0.26 to 0.64)	0.084 (0.002 to 0.77)	47
* Missing*		28		2		9		3			
LCQ Autonomy scale^f^	25.9 (6.4)	48	24.7 (7.1)	17	26.5 (6.9)	51	29.1 (5.0)	16	− 2.17 (− 7.12 to 2.78)	0.178 (0.01 to 0.80)	47
* Missing*		24		6		10		1			
LCQ Competence scale^f^	23.7 (6.1)	47	24.4 (6.3)	16	24.5 (6.4)	54	28.0 (5.3)	15	− 3.94 (− 8.23 to 0.35)	0.028	49
*Missing*		25		7		7		2			
LCQ Relatedness scale^f^	24.6 (7.4)	47	24.7 (6.7)	17	25.1 (7.4)	48	27.4 (6.4)	15	− 1.68 (− 6.83 to 3.48)	0.027	44
*Missing*		25		6		13		2			
Average daily minutes spent sedentary	518.1 (68.8)	47	508.2 (65.4)	11	469.9 (71.2)	34	567.9 (89.9)	6	− 87.0 (− 200.7 to 26.6)	0.291 (0.004 to 0.98)	30^d^
*Missing*		25		12		27		11			
									**Adjusted odds ratio**^**g**^** (95% CI)**		
School sport club/PA at least 3/week	10.5	57	9.5	21	31.6	57	35.3	17	1.16 (0.29 to 4.63)	0.018	63
*Missing*		15		2		4		0			
Non-school sport club/PA at least 3/week	27.9	61	35.0	20	42.1	57	35.3	17	1.07 (0.27 to 4.17)	0.006	66
*Missing*		11		3		4		0			

*SD* standard deviation, *CI* confidence interval, *ICC* intra-cluster coefficient, *M* mean, *SD* standard deviation, *MVPA* moderate to vigorous physical activity, *PAQ*-*C* self-reported physical activity, *LCQ* Learning Climate Questionnaire, *PA* physical activity

^a^Adjusted for baseline outcome and school. Mean differences are intervention minus control. A positive difference favours intervention; a negative difference favours Control

^b^95% confidence interval for some ICCs could not be estimated; assented to accelerometer at follow-up *n* = 77 (intervention group *n* = 62; control group *n* = 15)

^d^Limited to those who had valid data at baseline and follow-up

^e^Score range 1 = less to 5 = more physical activity

^f^Score range 6 = less, e.g. autonomy to 36 more autonomy

^g^Adjusted for baseline outcome and school. Odds ratio compares intervention to control as a ratio: OR > 1 favours intervention; < 1 favours Control

^h^Intervention group only at 12 months follow-up

#### Health economic evaluation

Complete case response rates for CHU-9D and EQ-5D-Y were 73.8% (*n* = 45 out of 61) and 92.6% (*n* = 25 out of 27) at baseline and 85.7% (*n* = 30 out of 35) and 88.4% (*n* = 38 out of 43) at follow-up, respectively. The percentage of pupils reporting maximum scores on EQ-5D-Y was 40.7% (*n* = 11 out of 27) at baseline and 48.8% (*n* = 21 out of 43) at follow-up compared to 6.6% (*n* = 4 out of 61) at baseline and 2.9% (*n* = 1 out of 25) at follow-up on CHU-9D. With respect to costing the intervention, it was possible to identify and measure the time spent on intervention set up and delivery from qualitative data collected via interviews with teachers and community role models.

#### Implementation of the intervention

Out of a possible 24 intervention sessions, 22 took place, with one cancellation due to adverse weather and another, to community role model illness. All session registers and observation forms were completed yet 50% of teacher observation forms had missing data. No adverse events were reported.

**Fig. 3:**
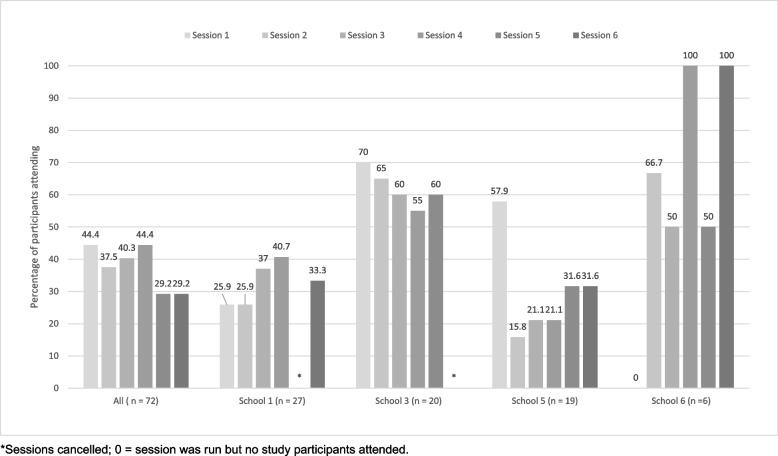
Percentage of participants in the intervention schools attending specific sessions, overall and by school

**Fig. 4:**
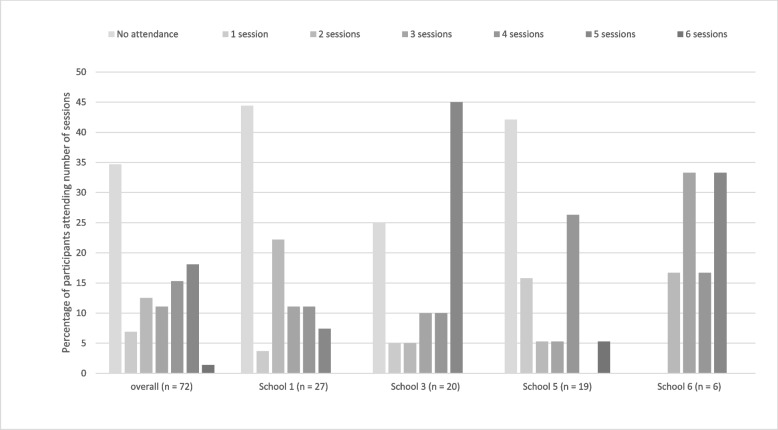
Percentage of participants in the intervention group attending total number of sessions

Session attendance fluctuated both within and across schools (Fig. 3). Of the 72 participants with parental consent for accelerometer wear and data collection, 47 (65%) attended at least one session with a median of two (25th to 75th centiles: 0.0 to 4.0) sessions per participant. This varied by school from a median of 1 session per participant (school 5) to 4 sessions per participant (school 3). Figure 4 shows the percentage of participants attending one and up to six sessions.

Intervention delivery was planned for the summer term of 2021 yet two schools chose to delay intervention delivery to the Autumn term due to significantly increasing school pressures during the Spring. Subsequently, delivery occurred during a time when the Omicron variant had high infection levels. Both schools reported consistently increasing coronavirus infection rates amongst pupils and staff, which considerably impacted upon intervention attendance. Historical challenges engaging with parents and families which could also have influenced challenges in attendance were noted at these two schools.

### Objective 3—acceptability of the intervention

#### Acceptability of the intervention

School staff, pupils and community and peer role models reported a positive experience of the intervention, with accounts of session enjoyment and opportunities for girls to try activities not typically provided at school. Peer role models described several personal benefits of their involvement including opportunities to; learn and lead activities, develop communication skills, build confidence, and support future career aspirations.

The importance of providing a girl-only intervention was recognised across all participant groups. Some boys said that there should be equity in opportunity and that girls do not get as much opportunity, yet some expressed a desire to take part in the intervention and study measures. School staff, community role models and peer role models highlighted positive and supportive interactions between girls during the sessions, with some girls modelling activities and giving friends and peers confidence to participate and try new skills. The girls-focused intervention was also described as aligning with both personal and organisational values of community role models and with school values and ethos.

School staff and girls generally considered community role models as knowledgeable, helpful and supportive. The involvement of peer role models in the intervention was also valued by pupils, school staff and most community role models, with community role models placing an emphasis on peers taking an active role in sessions. The relationship and role dynamic between community role models and peer role models was however an area highlighted for further refinement. The perceived relatability of peer role models to younger girls was apparent among teacher and community role model accounts, while some girls described how seeing the peer role models perform activities gave them the confidence to do it themselves. The design of the intervention, specifically linking to adjoining secondary schools was noted as highly beneficial for supporting girls throughout the transition years via opportunities to build relationships with secondary school teachers and pupils.

#### Variation in intervention implementation by school context

Data suggest that intervention delivery was largely consistent across all schools and in line with the core intervention components, although some between-schools differences were observed. Observation data indicate that session duration ranged between 50 and 60 min, most sessions (85%) included an opportunity for questions and answers, and signposting (within 77% of sessions) was largely through verbal means.

Most of the session activities delivered were positively viewed and deemed appropriate for the girls and for all but one session, teacher and researcher observations reported that all girls were engaged throughout the sessions. Qualitative and observation data also suggest that those delivering the intervention adhered to the theoretical underpinning of the study in terms of providing support for psychological needs of relatedness and competence [34].

The number of peer role models attending each session ranged between one to eight pupils and on four occasions no peer role models attended. Absences were due to teacher illness (two sessions), an after-school sports fixture clash and miscommunication between schools. For most sessions, peer role models were reported as participating in the session and interacting with the girls. Engagement was described as improving over time, as peer role models had an opportunity to build relationships with the girls.

#### Objective 4—extent to which each progression criteria are met

Three of the five criteria for progressing to a full-scale evaluation were met (implementation, acceptability and completion of primary outcome), with two criteria (2 and 5) unmet (see Table 1). Progression criterion 2 was heavily impacted by the COVID-19 pandemic and a few uncertainties related to attendance eligibility. The amber progression criteria for criterion 5 indicated the need for Trial Steering Committee (TSC) discussion about the feasibility of proceeding to a full-scale trial. In discussion with the TSC, it was agreed that a full-scale trial with an embedded optimisation period (e.g. 6–12 months prior) was warranted.

## Discussion

The primary aim of this study was to assess the feasibility and acceptability of CHARMING and its proposed evaluation methodology, to inform future evaluation decisions. Findings showed that it is possible to deliver an after-school community linked role model physical activity programme, using a primary-secondary school dyad design. The study design and intervention were acceptable; however, there were some challenges with participant recruitment and accelerometer wear time. Several avenues for future optimisation were identified.

Despite the challenges of the COVID-19 pandemic, it was possible to recruit schools to deliver the CHARMING intervention and to retain them within a cRCT study. While demonstrating a recruitment rate of 98% among approached girls, similar to wider school-based studies [46, 47], a large proportion of girls did not have parent consent to participate in the study. Opt-in consent can present a barrier to a child’s participation within a health promoting programme, often presenting more participation barriers among those in greatest need [48]. Qualitative data suggested however, that an opt-in consent approach was acceptable to schools therefore future work needs to identify processes for engaging with parents to ensure receipt of study information and provide multiple opportunities for addressing any research concerns.

Regarding the trial methodology, we showed that the design and methods are feasible for a larger trial. Accelerometer return rates exceeded 96%, with only three unreturned monitors at follow-up and survey participation was high among those with parent consent. Compliance with accelerometer wear time however was low, with only 57–64% of monitors yielding valid data. Considering the high percentage of girls consenting to wear the accelerometer and the low numbers with missing accelerometer data (i.e. not wearing the monitor), results suggest further strategies (e.g. in-class and parent reminders) or using alternative devices (i.e. wrist worn devices) to enhance wear time in a future full-scale trial are needed.

It was feasible to deliver an after-school community linked intervention to Year 5 girls. This was demonstrated amidst the challenging context of the COVID-19 pandemic which limited the length of the intervention and on reflection, schools and community role models expressed a desire for the intervention to run over a longer period in future (a 12-week intervention was originally proposed within the funding application). While a 6-week intervention was only possible in the given unique context, findings suggest that a longer intervention would be acceptable and feasible in the absence of a pandemic. A major consequence of the implementation context was seen in intervention attendance numbers. Teacher accounts confirmed the contextual challenges of COVID-19 isolation cases hindering intervention implementation and attendance rates, challenges noted by a wider peer-led school-based study [49]. Qualitative data indicated that pupils, school staff, peer- and community role models perceived the intervention to be acceptable, with positive accounts of involvement and a desire to take part in future work. This was also highlighted by the willingness of the 16 community role models to volunteer time and resource to be involved.

Results of the health economic analyses indicate that it is feasible to collect HRQoL information from 9- to 10-year-old girls using CHU-9D and EQ-5D-Y, with both having complete case response rates of over 70%. The high percentage of participants reporting maximum scores, indicating full health, on EQ-5D-Y, compared to CHU-9D indicates that EQ-5D-Y is more prone to ceiling effects, i.e. EQ-5D-Y appears less sensitive for determining differences between girls reporting health states approaching full health. Ceiling effects in EQ-5D-Y have been reported by other studies [50]. The qualitative data suggest it is feasible to identify and measure resource use for the purpose of costing the CHARMING intervention (i.e. time spent on intervention set-up and delivery) using interviews with teachers and community-role models. Further detail on the health economic analyses will be available in a separate paper.

The intervention design aimed to facilitate connections between primary and secondary schools and support pupil transition into secondary school. This key transition phase has been shown to coincide with declines in total physical activity levels [51–53]. The intervention design was perceived as highly beneficial for supporting pupils in their final years at primary school and building early relationships with secondary school teachers and older peers. Future optimisation and implementations of CHARMING will explore the potential for delivery elements to be based at the secondary school premises, as suggested by school staff, primary school girls and peer role models.

Regarding the newest intervention component, involving peer role models, this was demonstrated to be feasible and acceptable with peer role models themselves reporting enjoyment of being involved in the intervention and highlighting several personal benefits. Pupils, teachers and community role models also relayed benefits of peer role model involvement, which have been described in greater detail elsewhere [31]. There is scope however, to refine the recruitment materials and role documentation to further enhance experiences within activity sessions, in particular providing role clarity in relation to that of community role models. Recommended steps to enhance the future implementation of the role model component have been detailed in a separate publication [31].

### Strengths and limitations

The design and conduct of this study are in line with the nature of feasibility studies, with findings highlighting important considerations for future work. Key strengths of the study include a robust mixed-methods design and analyses, extensive process evaluation measures and the use of a novel intervention approach (i.e. primary-secondary dyad design with use of multiple role models). There are however several limitations to acknowledge. Randomisation resulted in a small number of participants allocated to the control group while no process evaluation data were collected from control schools, which prevented any understanding of randomisation acceptability among this group. Only two parents participated in interviews and as such the study provides limited depth and diversity of parent views and experiences. Data obtained from boys’ focus groups were also limited due to recruitment challenges within two schools. As the study took place amidst the COVID-19 pandemic, several strategies were implemented to support schools in implementing the study (i.e. increased administration support) and delivering the intervention (e.g. supporting community role model recruitment and facilitating delayed intervention delivery).

## Conclusions

CHARMING represents a novel co-produced intervention, which adopts a primary-secondary dyad approach and local community involvement. The current study demonstrates that the CHARMING intervention and cRCT design are both acceptable and feasible. Qualitative data provide several suggestions to support future improvements to optimise both the intervention and the evaluation design prior to a full scale cRCT.

## Supplementary Information

Additional file 1. Topic guide questions.

Additional file 2.

## Data Availability

The datasets generated during the current study are not currently publicly available due to wider publication plans.

## Abbreviations

CHARMING: CHoosing Active Role Models to INspire Girls
CI: Confidence Interval
CONSORT: Consolidated Standards of Reporting Trials
cRCT: Cluster Randomised Controlled Trial
CSRI: Client Service Receipt Inventory
ICC: Intra-Class Correlation
LA: Local authority
LSOA: Lower super output area
MVPA: Moderate-to-vigorous physical activity
NICE: National Institute for Health and Care Excellence
OR: Odds ratio
PAQ-C: Physical Activity Questionnaire for Older Children
PC: Progression criteria
SDT: Self-determination theory
TSC: Trial Steering Committee
UK: United Kingdom
WHO: World Health Organisation
