# Supplementary material for: Findings from a cluster randomised feasibility study of a school-based physical activity role model intervention (CHARMING) for 9–10-year-old girls
Source: Pilot Feasibility Stud. 2025 Apr 4;11:38. doi: 10.1186/s40814-025-01615-7 (PMC11969858; doi:10.1186/s40814-025-01615-7)
Supplement: Supplementary file 1 — Additional file 1. Topic guide questions. [file 40814_2025_1615_MOESM1_ESM.docx]

**Additional file 1 - Topic guide questions**

**Year 5 girls**

| 1. What did you think about wearing the activity monitor? |
| --- |
| 1. What were they like to wear? |
| 1. If you didn’t wear an activity monitor, are there any reasons why you decided not to? |
| 1. There was a paper diary with the activity monitor, and we asked girls to write down the times they took the activity monitor on and off. How did you find this? Did you manage to fill out the diary and bring it back to school? Anything we could do to make this better? |
| 1. How did you find filling out the survey? Did you find the questions easy/hard to understand? Anything we could do to make this better? |
| 1. Why did you want to take part in the CHARMING programme? |
| 1. What did you think about the CHARMING programme? |
| 1. What activities did you most enjoy? Can you tell me why you enjoyed this activity the most? |
| 1. Did you get to try new activities you hadn’t tried before? |
| 1. Was there anything you didn’t like about the programme? |
| 1. Was there anything else you wish you could have done? |
| 1. How much choice did you have about what activities you did? Would you have liked more/less choice? |
| 1. What helped you to keep taking part in the activities every week? (Parents, teachers, peers etc.) |
| 1. Was it to do with the type of activities planned? |
| 1. Did you keep taking part because you liked the activity? |
| 1. Did it help to have a different activity every week? Would you have liked to do more of the same activity? |
| 1. Was it because you got to try a new activity? How do you find this? What was that like? |
| 1. If you didn’t try anything new, would you have liked to? |
| 1. The programme was delivered after school finished, do you think this is the best time or should it be at another time in school? |
| 1. What did you think of the people leading the sessions (community role models)? |
| 1. What did you like about these people? (I.e., qualities or characteristics - age, skills, experience, sporting ability?) |
| 1. Was there anything you didn’t like about them? |
| 1. Did you get to talk to them and find out what they do? Were you able to ask questions? How did you find this? |
| 1. What did you think of the peer role models? |
| 1. What did you like about these people? (I.e., qualities or characteristics - age, skills, experience, sporting ability?) |
| 1. What kind of things did they do during the sessions? What could they have done differently? |
| 1. What did you think about the number of peer role models at the sessions? Should there be less or more? |
| 1. Was it the same people every week or did it change? Should they stay the same every week? |
| 1. Was there anything you didn’t like about them? |
| 1. What did you think about the sessions being for girls only? |
| 1. Are you going to join any clubs/continue with any activities that you tried in the CHARMING Programme? (Why/why not?) |
| 1. How would you feel about the CHARMING Programme being longer (e.g., for 12 weeks instead of 6)? |

**Year 5 boys**

| 1. What types of physical activity, sports or exercise do you like to take part in? |
| --- |
| 1. Do you attend any sports/physical activity clubs at school or in your free time? What types of activities are these? |
| 1. Do you think girls like different sports and physical activity compared to boys? (If yes: Why do you think that may be?) |
| 1. How do you think we could help girls to be more active and to continue enjoying physical activities? |
| 1. How do you feel about the programme being just for girls? |
| 1. Are there any clubs in your school or in the area where you live which are for boys only? |
| 1. As part of our study, we asked girls in Year 5 to complete a survey and wear an activity monitor (belt they wore around their waist). We would like to hear what you thought about only the girls being asked to do these? |

**Teachers / Head teachers**

| **Question** |
| --- |
| 1. How long have you been in your current role? |
| 1. Can you tell me about your understanding of the CHARMING programme, what it is, what it involves, what it aims to do? |
| 1. Can you tell me about your school’s approach to health and well-being? |
| 1. Other than CHARMING, what other physical activity programmes/initiatives have you been involved with in your school? |
| 1. How does the CHARMING programme align with your school’s approaches and ethos to PA promotion? |
| 1. In a way, the CHARMING programme has an element of support for transition between primary and secondary school, what other things does your school do to support transition? How does CHARMING fit with this, or differ to this? |
| 1. Can you tell me about the role you have played in your school’s involvement in the CHARMING study? |
| 1. Why did you decide to join/agree to join the study? (£200 incentive) |
| 1. Did you meet with (a. Parents and b. Other Teaching staff) to discuss the CHARMING programme and your involvement? If yes, how many hours/mins did you spend discussing the CHARMING intervention with parents? |
| 1. Did you liaise with the trainer(s)/coach(es) to discuss PA sessions as part of the CHARMING intervention ? If yes, how many hours/mins did you spend liaising with them? |
| 1. Can you tell me what you know about the data collection processes involved in the study? |
| 1. What do you know about the experience of implementing the CHARMING Programme within your school? |
| 1. What did you think about the quality and quantity of information you received throughout the study, if any? |
| 1. Was there ever a point during which you or others in the school wanted to stop doing the study? |
| 1. The programme was delivered after-school. Has this ‘fitted in’ with the school timetable and school structure? |
| 1. How does CHARMING fit within usual practice? |
| 1. Do you think the CHARMING programme has the potential to increase or sustain Year 5 girls’ physical activity? |
| 1. If it is shown to be effective, would your school be interested in delivering the programme in future? |
| 1. What are your thoughts on running the programme for a longer period, for example for 12 weeks? |
| 1. If CHARMING was to be rolled out to all primary schools who should be responsible for coordinating it / funding it? |
| 1. Do you have any other thoughts about the impact of COVID-19 on implementing (a. the research study and b. the programme)? |
| **Specific questions for the lead primary school teacher** |
| 1. How was your experience setting up the delivery of the programme? |
| 1. How do you think the girls found taking part in the programme? |
| 1. How did girls’ engagement change over time? |
| 1. Do you think there could be any unintended/negative impacts of the programme? |
| 1. What was it like having the external community role models (coaches) attend the school? |
| 1. Did you liaise with the secondary school in relation to the peer role models? |
| 1. What do you understand about the role that peer role models play in the programme? Why do you think peer role models are involved in this programme for primary school pupils? |
| 1. What was it like delivering the programme after school? |
| 1. Have you received any other feedback from pupils, parents, and teachers? |

**Community role models**

| 1. What is your job title? What organisation or club are you linked to? |
| --- |
| 1. How long have you been in your current role? |
| 1. What is your highest qualification (BTEC, BSc, MSc, etc )? |
| 1. Tell me a bit about yourself in relation to your coaching experience and your sport/community club? |
| 1. Can you tell me how you heard about this opportunity? |
| 1. Why did you decide to deliver a taster session? |
| 1. How could we advertise this opportunity more widely? |
| 1. How many hours/mins did you spend planning your PA session(s) for the CHARMING Programme (including booking a space to run it)? |
| 1. Did you adapt your delivery of the CHARMING Programme session(s) to deal with COVID-19 restrictions/ delivery outdoors? |
| 1. The CHARMING Programme sessions were planned as 1-hour slots, did you need to arrive early to set up the venue? |
| 1. The CHARMING Programme sessions were planned as 1-hour slots, did you need to stay late to clean up the venue? |
| 1. Did you meet/speak with school staff to discuss the CHARMING Programme and your involvement? |
| 1. On average, how far did you travel to and from (round trip) the CHARMING Programme session venue in miles? |
| 1. What mode of transport did you use to travel (e.g., walk, car, bus, lift share) to the (majority of the) PA session(s)? |
| 1. Did you purchase any equipment in order to deliver the CHARMING Programme sessions? |
| 1. Can you tell me a bit about the activity you delivered for the CHARMING Programme session? |
| 1. How do you think the primary school girls found taking part in your session(s)? |
| 1. What kind of things did you do during the activity to encourage and motivate the girls to engage and participate? |
| 1. How do you feel the pupils interacted with you during the session activity? |
| 1. The CHARMING programme includes the involvement of secondary school students to support primary school pupils’ engagement in physical activity and to act as ‘peer role models’. Were there peer role models present in your session(s)? |
| 1. Reflecting on how your session went, was there anything you would do differently next time? |
| 1. Have you received any other feedback from pupils, parents, and teachers? |
| 1. What about the quality and quantity of information you received about the study/CHARMING Programme and what was required from your involvement? |
| 1. Was there ever a point during which you wanted to stop doing the study/CHARMING Programme? |
| 1. Can you tell me about how the CHARMING programme fits with the priorities of your organisation and its work around physical activity promotion? |
| 1. What were the main challenges in organising/coordinating/implementing the CHARMING Programme, i.e., ‘making it happen’? |
| 1. What things helped with delivering the CHARMING Programme? |
| 1. Would you recommend it to other coaches? |
| 1. If the CHARMING Programme was to be rolled out to all primary schools in your area, e.g., over the course of a whole school year, would your organisation/club be interested in delivering similar sessions across this larger range of schools? |
| 1. What was the impact of COVID-19 on delivering the CHARMING Programme session? |

**Peer role models**

| 1. How did you hear about the opportunity to be a peer role model in CHARMING? |
| --- |
| 1. What other things do they/their school do with their feeder primaries? |
| 1. What did you think about the information you were given? |
| 1. Thinking back to when you first heard about CHARMING, did you understand what you would have to do to be a peer role model? |
| 1. What did your parents/carer think? |
| 1. Why did you decide to take part? What happened next? |
| 1. What did you think about attending the primary school for the CHARMING Programme? |
| 1. What was it like travelling to the primary school? |
| 1. Were there any times during the study where you couldn’t attend the CHARMING Programme? |
| 1. In this primary school, CHARMING is being delivered for 6 weeks, however some research suggests it might be better if it was longer, perhaps 12 weeks (3 months). What do you think of that? |
| 1. How about, if CHARMING was delivered to all of the primary schools in your area, would you be interested in being a peer role model at more than one school? |
| 1. What did being a peer role model involve? |
| 1. What did you think about being a peer role model in the CHARMING Programme? What was it like? |
| 1. What do you think makes someone a good peer role model for the CHARMING programme? |
| 1. What did you gain from the experience? |
| 1. Why do you think that the CHARMING programme involves peer role models? |
| 1. Is there anything that you would change about the peer role models participating in the CHARMING Programme? |
| 1. The sessions were delivered after school finished; do you think this is the best time for the CHARMING Programme? |
| 1. Did you speak to your friends/parents/carers about the CHARMING Programme sessions? |
| 1. What did you think about running the CHARMING Programme during COVID-19? |
